# Missed Opportunities for Compassion: A Call to Action for Incarcerated Individuals Denied Compassionate Release

**DOI:** 10.1093/geroni/igab046.3218

**Published:** 2021-12-17

**Authors:** Claire Morton, Rachel Nathan, Anjana Chacko, Raya Kheirbek

**Affiliations:** 1 Une, Baltimore, Maryland, United States; 2 University of Maryland School of Medicine, University of Maryland, Maryland, United States; 3 University of Maryland, Baltimore, Maryland, United States; 4 University of Maryland School of Medicine, University of Maryland School of Medicine, Maryland, United States

## Abstract

In 2016, a total of 4,117 state and federal prisoners died in publicly or privately operated prisons. Each year from 2001 to 2016, an average of 88% of deaths in state prisons were due to natural causes, with more than half of those due to cancer, heart disease or liver disease, conditions for which non-incarcerated citizens often benefit from palliative care and hospice. Prisoners age 55 and older are the fastest-growing segment of the population residing in prisons, as well as those with the highest mortality rate. Compassionate release of seriously ill prisoners became a matter of federal statute in 1984 and has currently been adopted by the majority of U.S. prison jurisdictions. The spirit of the mandate is based on the idea that catastrophic health conditions ie terminal illness affect the four principles of incarceration: retribution, rehabilitation, deterrence, and incapacitation. Concerned about an aging prison population, overcrowded facilities, and soaring costs, many policy makers are calling for a wider use of compassionate release for persons with terminal illness as well as broader prison reform. The prognosticating criteria of compassionate release guidelines are clinically flawed, and the application and procedural barriers are prohibitive. In this paper we review cases of patients who qualified for compassionate release but had their applications denied. We will discuss the urgent need for access to quality palliative medicine for incarcerated persons with advanced illness and call healthcare providers to action with the aim of reducing suffering and promoting social justice for those in need.

